# Cardiovascular effects of high-fructose intake in rats with nitric oxide deficiency

**DOI:** 10.2478/intox-2014-0022

**Published:** 2014-12-30

**Authors:** Anna Zemančíková, Jozef Török

**Affiliations:** Institute of Normal and Pathological Physiology, Slovak Academy of Sciences, Bratislava, Slovak Republic

**Keywords:** fructose, nitric oxide, hypertension, conduit artery

## Abstract

The aim of this study was to evaluate the involvement of nitric oxide (NO) system damage in the deleterious effects of high-fructose intake in rats. Fructose was administered as 10% solution in drinking water to twelve-week-old male Wistar rats for the period of 8 weeks. Blood pressure was measured by tail-cuff plethysmography. After sacrificing the rats at the end of the treatment, relative weights of heart and liver and biochemical parameters in blood plasma were determined. Reactivity of isolated conduit arteries was measured using a force-displacement transducer for recording isometric tension. Fructose drinking rats had increased blood pressure and impaired acetylcholine-induced relaxation of the thoracic aorta in comparison with control rats drinking just tap water. Relative liver weight and plasma concentrations of glucose and triglycerides were also elevated after fructose administration. In a further group of Wistar rats, inhibition of NO production by administration of N^G^-nitro-L-arginine methyl ester (L-NAME; 40 mg/kg/day) was performed throughout fructose intake. L-NAME treatment itself induces increase in blood pressure and relative heart weight as well as impairment in arterial relaxation and contractility. However, in these rats, fructose administration did not cause further elevation of blood pressure and other abnormalities observed in rats receiving fructose without L-NAME. Our results showed that in the state of NO deficiency (induced by L-NAME administration) fructose does not induce cardiovascular and metabolic alterations which develop in rats with a functional NO system. This indicates that impairment of the NO system may participate in many of the adverse effects induced by high-fructose intake.

## Introduction

Nitric oxide (NO) plays a critical role as a molecular mediator in a variety of biological processes, including vasodilatation, neurotransmission and macrophage-mediated immunity. In spite of its pleiotropic effects in organisms, its role has been most extensively studied in physiology and pathology of the cardiovascular system. It is its prominent role in endothelium-dependent relaxation of vessels thanks to which the function of NO in the cardiovascular system was discovered and identified (Furchgott & Zawadzki, 1980; Ignarro *et al*., 1987). In vascular endothelium, as well as in other cells of the body, NO is synthesized from L-arginine in a reaction catalyzed by nitric oxide synthase (NOS). Blockade of this enzyme in experimental conditions by administration of arginine analogues (*e.g*. N^G^-nitro-L-arginine methyl ester – L-NAME) causes NO deficiency and impaired vascular relaxation leading to sustained blood pressure increase (Gardiner *et al*., 1990; Török *et al*., 1998).

Alterations in synthesis and availability of NO may play an important role also in various cardiovascular dysregulations occurring in clinical conditions in humans. Decrease in the amount of NO due to various mechanisms is often considered one of the conditions leading or contributing to the development of genetic or environmentally induced hypertension. Many factors associated with modern lifestyle may evoke sustained blood pressure elevation or increase the probability of its manifestation in predisposed individuals, which has been confirmed epidemiologically as well as in experimental conditions (Beilin, 1999; Bernatova *et al*., 2010). Changes in food habits and food composition during the last decades may represent one of these factors. Increased consumption of added sugars, especially fructose (as monosaccharide or bound to glucose in sucrose) has been the focus of much interest as a possible contributor to the current epidemic of obesity and diabetes-related diseases, including hypertension (Elliott *et al*., 2002; Tappy & Lê, 2010).

Fructose has a unique metabolism occurring predominantly in the liver and it is more lipogenic than that of glucose (Havel, 2005). This property may have detrimental consequences when fructose is consumed in excess for a long period. In experimental conditions, it was confirmed many times that rats receiving high amount of fructose (in diet or in drinking water) during several weeks became hypertriglyceridemic, insulin resistant and hyperinsulinemic. In addition, these individuals often exhibited many other abnormalities like hepatic steatosis, obesity and hypertension, although there are some discrepancies in these findings. Detailed characteristics of fructose metabolism and its possible link with cardiometabolic disorders have been the subject of several reviews (Havel, 2005; Tappy & Lê, 2010).

Involvement of NO and its reduced availability in high-fructose-induced damage has been indicated in several studies (Miatello *et al*., 2001; Takagawa *et al*., 2001). However, it is not clear whether impairment of the NO system is crucial in triggering the development of these processes. It might be supposed that particularly the pro-hypertensive effect of high-fructose intake is substantially evoked by the decrease in NO amount within cardiovascular tissues. Thus the main goal of this study was to compare the effects of fructose administration on some cardiovascular and metabolic parameters in normal and NO deficient rats and to evaluate the involvement of NO system damage in the deleterious effects of high-fructose intake.

## Methods

### Experimental animals

Twelve-week-old male Wistar rats were housed at 22–24°C on a 12:12-h dark-light cycle (06.00–18.00 h lights on) and maintained on a standard laboratory rat chow *ad libitum*. They were randomly divided into four groups: control rats (drinking tap water), fructose drinking rats (receiving 10% solution of fructose for 8 weeks), L-NAME treated rats (receiving 40 mg/kg/day of L-NAME in drinking water for 8 weeks), L-NAME treated fructose drinking rats (receiving 40 mg/kg/day of L-NAME in 10% solution of fructose for 8 weeks). The animal protocols used in this study were performed in accordance with the Guide for the Care and Use of Laboratory Animals published by the National Institutes of Health, and approved by the Animal Health and Welfare Division of the State Veterinary and Food Administration of the Slovak Republic.

Systolic blood pressure and heart rate were measured in conscious rats by the non-invasive tail-cuff method. At the end of the experiment (in the 20^th^ week of life), all rats were fasted overnight (in the group of fructose drinking rats, fructose solution was replaced by tap water) and then they were sacrificed under CO_2_ anesthesia. Samples of their blood were collected immediately and used for measurement of plasma glucose, cholesterol and triglyceride concentration. Wet weight of the left heart ventricle and of the liver and the length of the tibia were determined for calculation of the ratio of left heart ventricle weight to tibia length and of liver weight to tibia length. The thoracic aorta and superior mesenteric artery were carefully removed and prepared for functional studies performed by isometric tension recording.

### Measurement of arterial reactivity *in vitro*

The arteries were cut into rings (3.0–3.5 mm in width) and suspended in 20 ml organ baths filled with oxygenated (95% O_2_ + 5% CO_2_) modified Krebs solution maintained at 37°C. The Krebs solution had the following composition (in mmol/l): NaCl 118, KCl 5, CaCl_2_ 2.5, MgSO_4_ 1.2, NaHCO_3_ 25, KH_2_PO_4_ 1.2, glucose 11, CaNa_2_.EDTA 0.03. The arterial rings were set up for isometric tension recording using a force-displacement transducer Sanborn FT 10 (Sanborn, Baltimore, USA). The preparations were equilibrated under a resting tension of 10mN for 60–90 min and the solution was changed every 15 min.

To examine the endothelium-dependent vasorelaxation, the preparations of the aorta were first precontracted by phenylephrine (10^–6^ mol/l). When the contraction reached a plateau, increasing concentrations of acetylcholine were applied in a cumulative manner (10^–9^–10^–5^ mol/l).

Adrenergic contractions were determined in endothelium-intact thoracic aortas and mesenteric arteries as the responses to cumulatively applied exogenous noradrenaline (10^–10^–3×10^–5^ mol/l).

In mesenteric arteries, neurogenic responses were induced by electrical stimulation of periarterial sympathetic nerves. The arterial rings were stimulated by two parallel platinum plate electrodes placed on either side of the preparation and connected to an electrostimulator ST-3 (Hungary). Frequency-response curves to electrical stimuli were obtained using square pulses of 0.5 ms in duration, at supramaximal voltage (>30 V), applied at 1–32Hz, for a period of 20 s. In our preliminary observations we found that the contractions of rat mesenteric arteries elicited by electrical stimulation (using the described parameters of stimulation) were blocked by phentolamine or tetrodotoxin, indicating that they were induced by nerve-released (endogenous) noradrenaline.

Acetylcholine chloride, L-Noradrenaline hydrochloride and *N* ^ω^-Nitro-L-arginine methyl ester hydrochloride (L-NAME) were purchased from Sigma-Aldrich (Germany); other chemicals were purchased from local commercial sources.

### Data analysis

The results are presented as means ± S.E.M. The arterial responses to particular pharmacological and electrical stimuli are expressed as absolute values in mN and normalized to the cross sectional area of the respective ring preparation.

Statistical evaluation was carried out by using one-way analysis of variance (ANOVA). The results were considered to be significant when *p*<0.05.

## Results

After eight weeks of treatment with fructose, the Wistar rats had significantly elevated blood pressure without change in heart rate, when compared to control untreated rats. Body weight and relative weight of the left heart ventricle did not differ between control and fructose drinking rats. However, the relative liver weight, as well as plasma glucose and triglyceride concentrations were increased due to fructose treatment (Table 1).

**Table 1:** Effect of 8-week-lasting high-fructose intake on some cardiovascular and metabolic parameters in control and L-NAME treated Wistar rats.

	Wistar	Wistar + fructose	Wistar + L-N	Wistar + L-N+ fructose
**BP** (mmHg)	118.1±2.0	124.6±1.6 *	151.2±4.4 †††	155.6±6.4
**HR** (bpm)	402.3±6.1	392.2±9.9	402.6±13.0	406.6±11.6
**BW** (g)	469.1±15.9	468.2±13.8	447.8±17.7	465.3±14.6
**LVW/BW** (mg/g)	2.01±0.04	2.01±0.10	2.33±0.10 ††	2.21±0.08
**LiW/BW** (mg/g)	35.32±0.85	41.69±0.61 ***	33.91±0.87	35.14±2.39
**GLU** (mmol/l)	10.32±0.73	13.8±0.79 *	8.73±0.89	7.16±0.46
**CHOL** (mmol/l)	1.69±0.07	1.83±0.11	1.98±0.10 †	2.25±0.30
**TRIGL** (mmol/l)	1.57±0.17	2.69±0.42 *	1.34±0.29	2.15±0.36

Abbreviations: L-N – N^G^-nitro-L-arginine methyl ester (L-NAME), BP – blood pressure, HR – heart rate, BW – body weight, LVW/BW – left ventricular weight-to-body weight, LiW/BW – liver weight-to-body weight, GLU – glucose, CHOL – cholesterol, TRIGL – triglycerides. Values represent mean±SEM; n = 5–8;

* *p*<0.05

*** *p*<0.001 Wistar *vs*. Wistar + fructose;

† *p*<0.05

†† *p*<0.01

††† *p*<0.001 Wistar *vs*. Wistar + L-N.

**Figure 1:**
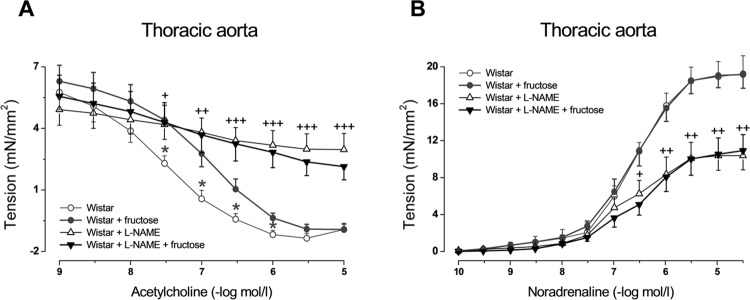
Effect of 8-week high-fructose intake on acetylcholine-induced relaxation (A) and noradrenaline-induced contraction (B) in aortic rings from control and L-NAME treated Wistar rats. Values represent mean±SEM; n = 5–8; *p<0.05 Wistar *vs*. Wistar + fructose; ^+^ *p*<0.05, ^++^ *p*<0.01, ^+++^ *p*<0.001 Wistar *vs*. Wistar + L-NAME.

**Figure 2:**
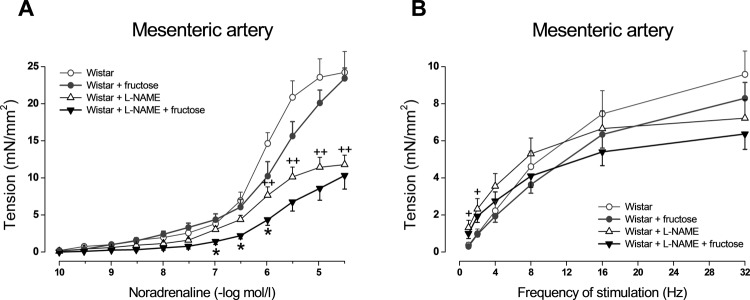
Effect of 8-week high-fructose intake on noradrenaline-induced contraction (A) and neurogenic contraction (induced by electrical stimulation of perivascular nerves) (B) in rings of superior mesenteric artery from control and L-NAME treated Wistar rats. Values represent mean±SEM; n = 5–8; *p<0.05 Wistar + L-NAME *vs*. Wistar + L-NAME + fructose; ^+^ *p*<0.05, ^++^ *p*<0.01 Wistar *vs*. Wistar + L-NAME.

In the thoracic aorta, endothelium-dependent relaxations in response to acetylcholine were significantly decreased after fructose administration (Figure 1). Contractile responses to exogenous noradrenaline as well as to endogenous noradrenaline released from perivascular nerves during electrical stimulation were not changed in fructose drinking rats in comparison with untreated rats (Figures 1 and 2).

Eight-week L-NAME administration to control Wistar rats caused significant increase in blood pressure, relative weight of left heart ventricle, and in plasma cholesterol level (Table 1). It also led to decrease in endothelium-dependent relaxations and noradrenergic contractions in the arteries examined (Figures 1 and 2). However, L-NAME treatment caused elimination of cardiovascular and metabolic changes induced by fructose: in L-NAME-treated rats, simultaneous fructose administration did not cause further elevation in blood pressure, relative liver weight, plasma glucose and triglyceride levels nor impairment of endothelium-dependent relaxation (Table 1, Figure 1). The only effect of fructose administration in L-NAME-treated rats was decrease in noradrenaline-induced contractions of mesenteric arteries (Figure 2).

## Discussion

In this study we found that eight-week-lasting fructose administration to adult Wistar rats induced metabolic changes associated with increase in plasma glucose and lipids, enlargement of liver, mild but significant blood pressure elevation, and reduction in acetylcholine-induced arterial relaxation. We also showed that in rats made NO deficient by L-NAME treatment, the high-fructose intake did not evoke such alterations. These observations indicate that impairment of the NO system might represent an important mechanism of high-fructose induced damage.

It was shown by other investigators that production of NO decreased after feeding normal rats with excess of fructose and that this effect may induce hypertension development in such treated individuals (Miatello *et al*., 2001; Takagawa *et al*., 2001). In fact, specific association of high-fructose consumption with elevation of blood pressure represents one of the controversial issues. Many studies found a clear pro-hypertensive effect of fructose administration (Hwang *et al*., 1987; Dai & McNeill, 1995; Verma *et al*., 1994; Miatello *et al*., 2001); however, other authors found no influence of this factor on blood pressure (Brands *et al*., 1994; D'Angelo *et al*., 2005). This discrepancy may be attributed to either dietary composition, differences in age or strain of experimental animals, or to the different techniques of blood pressure measuring (Ferrari *et al*., 1990; Johnson *et al*., 1993; Brands *et al*., 1994).

It is known that even after excessive intake of fructose, its peripheral plasma concentration remains quite low due to immediate absorption and degradation in the liver (Tappy & Lê, 2010). It is therefore reasonable to suppose that the possible pro-hypertensive effect may be caused by products of fructose hepatic metabolism. One such compound is uric acid which is highly produced during fructose catabolism in hepatocytes. It was shown that elevated plasma concentration of uric acid may contribute to the development of type 2 diabetes and hypertension in subjects with metabolic syndrome (Johnson *et al*., 2009; Perez-Pozo *et al*., 2010). The mechanisms appear to involve the reduction of NO bioavailability in endothelial cells, adipocytes, and vascular smooth muscle cells due to oxidative stress (Khosla *et al*., 2005; Sautin *et al*., 2007; Corry *et al*., 2008), stimulation of arginase (Zharikov *et al*., 2008), and the direct scavenging of NO by uric acid (Gersch *et al*., 2008). Moreover, Corry *et al*. (2008) demonstrated that uric acid stimulated proliferation and angiotensin II production in vascular smooth muscle cells. The described effects are responsible for endothelial dysfunction and prevalence of vasoconstriction, leading to increase in vascular resistance and in blood pressure. Incapability of generating endothelial NO and reduced blood flow to skeletal muscle and peripheral tissues deteriorate insulin action and glucose uptake by the cells and may lead to insulin resistance. Compensatory hyperinsulinemia can stimulate sympathetic nervous system activity and kidney sodium reabsorption, which also promote elevation of blood pressure (Corry *et al*., 1999).

In the present experiments we observed elevation of blood pressure along with decreased acetylcholine-induced relaxation of the aorta in fructose drinking rats, yet these alterations were not found when NO production was eliminated by L-NAME. This may demonstrate the high level of NO dependency of fructose-induced influence on these parameters. In such a situation no additive effect of fructose and L-NAME administration should be supposed because the dose of L-NAME used in our treatment is sufficiently high to strongly inhibit NO production (Pechánová *et al*., 1999).

Different results were obtained in spontaneously hypertensive rats (SHR) which exhibit similar severity of hypertension as L-NAME-treated rats and their arteries have also diminished endothelium-dependent relaxation. However, in conduit arteries of adult SHR the NO synthase activity is markedly elevated and their resting relaxant capacity is still sufficiently NO dependent (Puzserova *et al*., 2014). Eight-week-lasting fructose administration to SHR led to further blood pressure increase and impairment of acetylcholine-induced relaxation (Török *et al*., 2012), supporting the idea that even in a severe hypertensive state, when there is sufficient NO production, the high-fructose-induced impairment of cardiovascular function might be manifested.

The presented observations show that fructose administration had no effect on the magnitude of arterial contractions induced by exogenous or endogenous noradrenaline. On the other hand, treatment of rats with L-NAME alone led to diminution of arterial contractile responses. This paradoxical effect of inhibiting NO synthase was demonstrated by many authors (Dowell *et al*., 1996; Zemančíková & Török, 2013). It was shown that besides the impairment of vasorelaxation and the consequent increase in the sensitivity of vascular smooth muscle to constrictoric stimuli, sustained inhibition of NO production has some additional impact on the cardiovascular system, including structural alterations or functional changes in vascular smooth muscle cells resulting from decreased contractile protein expression and reduction in extracellular calcium influx, which may lead to loss of their contractile properties (López *et al*., 1996; Yoneyama *et al*., 1998). We observed that during treatment with NO synthase inhibitor, simultaneous high-fructose intake surprisingly induced further decrease in contractile force development of the mesenteric artery in response to exogenously applied noradrenaline. This finding may suggest that high-fructose intake may induce some unknown NO-independent mechanism involved in the reduction of contraction of the mesenteric artery, which was not seen in preparations of the thoracic aorta.

Inhibition of NO production eliminated also the high-fructose evoked increase in liver mass and in plasma glucose and lipids. It is known that NO is implicated in a myriad of mechanisms regulating liver metabolism and its higher amount may have the potential for both protecting the liver as well as exacerbating its injury. Spruss *et al*. (2011) demonstrated that inducible NO synthase (iNOS) is involved in the onset of fructose-induced liver steatosis and that the key factor in this process is the increased formation of reactive oxygen species in hepatic tissue. In mice receiving high amounts of fructose (Spruss *et al*., 2011) or in rats with cholestasis (Monsef, 2012), various parameters indicating the status of inflammation and fibrosis in liver were ameliorated after treatment with unspecific NO synthase inhibitors like L-NAME, or in iNOS knockout mice. This may partially elucidate our observation of the failure of fructose administration to induce abnormalities in liver and plasma parameters in NO deficient rats.

In conclusion, in this study we demonstrated that NO deficiency abolished the abnormalities observed during fructose administration in normal rats. It may thus be supposed that impairment of the NO system may participate in many of the adverse effects induced by high-fructose intake since a functional NO system is necessary for their manifestation.

## References

[CIT0001] Beilin LJ (1999). Lifestyle and hypertension--an overview. Clin Exp Hypertens.

[CIT0002] Bernatova I, Puzserova A, Dubovicky M (2010). Sex differences in social stress-induced pressor and behavioral responses in normotensive and prehypertensive rats. Gen Physiol Biophys.

[CIT0003] Brands MW, Garrity CA, Holman MG, Keen HL, Alonso-Galicia M, Hall JE (1994). High-fructose diet does not raise 24-hour mean arterial pressure in rats. Am J Hypertens.

[CIT0004] Corry DB, Eslami P, Yamamoto K, Nyby MD, Makino H, Tuck ML (2008). Uric acid stimulates vascular smooth muscle cell proliferation and oxidative stress via the vascular renin-angiotensin system. J Hypertens.

[CIT0005] Corry DB, Tuck ML (1999). Obesity, hypertension, and sympathetic nervous system activity. Curr Hypertens Rep.

[CIT0006] D'Angelo G, Elmarakby AA, Pollock DM, Stepp DW (2005). Fructose feeding increases insulin resistance but not blood pressure in Sprague-Dawley rats. Hypertension.

[CIT0007] Dai S, McNeill JH (1995). Fructose-induced hypertension in rats is concentration- and duration-dependent. J Pharmacol Toxicol Methods.

[CIT0008] Dowell FJ, Henrion D, Duriez M, Michel JB (1996). Vascular reactivity in mesenteric resistance arteries following chronic nitric oxide synthase inhibition in Wistar rats. Br J Pharmacol.

[CIT0009] Elliott SS, Keim NL, Stern JS, Teff K, Havel PJ (2002). Fructose, weight gain, and the insulin resistance syndrome. Am J Clin Nutr.

[CIT0010] Ferrari AU, Daffonchio A, Albergati F, Bertoli P, Mancia G (1990). Intra-arterial pressure alterations during tail-cuff blood pressure measurements in normotensive and hypertensive rats. J Hypertens.

[CIT0011] Furchgott RF, Zawadzki JV (1980). The obligatory role of endothelial cells in the relaxation of arterial smooth muscle by acetylcholine. Nature.

[CIT0012] Gardiner SM, Compton AM, Bennett T, Palmer RM, Moncada S (1990). Regional haemodynamic changes during oral ingestion of NG-monomethyl-L-arginine or NG-nitro-L-arginine methyl ester in conscious Brattleboro rats. Br J Pharmacol.

[CIT0013] Gersch C, Palii SP, Kim KM, Angerhofer A, Johnson RJ, Henderson GN (2008). Inactivation of nitric oxide by uric acid. Nucleosides Nucleotides Nucleic Acids.

[CIT0014] Havel PJ (2005). Dietary fructose: implications for dysregulation of energy homeostasis and lipid/carbohydrate metabolism. Nutr Rev.

[CIT0015] Hwang IS, Ho H, Hoffman BB, Reaven GM (1987). Fructose-induced insulin resistance and hypertension in rats. Hypertension.

[CIT0016] Ignarro LJ, Buga GM, Wood KS, Byrns RE, Chaudhuri G (1987). Endothelium-derived relaxing factor produced and released from the artery and vein is nitric oxide. Proc Natl Acad Sci USA.

[CIT0017] Johnson MD, Zhang HY, Kotchen TA (1993). Sucrose does not raise blood pressure in rats maintained on a low salt intake. Hypertension.

[CIT0018] Johnson RJ, Perez-Pozo SE, Sautin YY, Manitius J, Sanchez-Lozada LG, Feig DI, Shafiu M, Segal M, Glassock RJ, Shimada M, Roncal C, Nakagawa T (2009). Hypothesis: could excessive fructose intake and uric acid cause type 2 diabetes?. Endocr Rev.

[CIT0019] Khosla UM, Zharikov S, Finch JL, Nakagawa T, Roncal C, Mu W, Krotova K, Block ER, Prabhakar S, Johnson RJ (2005). Hyperuricemia induces endothelial dysfunction. Kidney Int.

[CIT0020] López RM, Ortíz CS, Ruíz A, Vélez JM, Castillo C, Castillo EF (2004). Impairment of smooth muscle function of rat thoracic aorta in an endothelium-independent manner by long-term administration of N(G)-nitro-L-arginine methyl ester. Fundam Clin Pharmacol.

[CIT0021] Miatello R, Risler N, Castro C, González S, Rüttler M, Cruzado M (2001). Aortic smooth muscle cell proliferation and endothelial nitric oxide synthase activity in fructose-fed rats. Am J Hypertens.

[CIT0022] Monsef A (2012). Effects of nitric oxide synthase inhibitor (L-NAME) on cytopathologic changes due to cholestasis in hepatic cells of adult male rats. Pol J Pathol.

[CIT0023] Pechánová O, Bernátová I, Pelouch V, Babál P (1999). L-NAME-induced protein remodeling and fibrosis in the rat heart. Physiol Res.

[CIT0024] Perez-Pozo SE, Schold J, Nakagawa T, Sánchez-Lozada LG, Johnson RJ, Lillo JL (2010). Excessive fructose intake induces the features of metabolic syndrome in healthy adult men: role of uric acid in the hypertensive response. Int J Obes (Lond).

[CIT0025] Puzserova A, Ilovska V, Balis P, Slezak P, Bernatova I (2014). Age-related alterations in endothelial function of femoral artery in young SHR and WKY rats. Biomed Res Int.

[CIT0026] Sautin YY, Nakagawa T, Zharikov S, Johnson RJ (2007). Adverse effects of the classic antioxidant uric acid in adipocytes: NADPH oxidase-mediated oxidative/nitrosative stress. Am J Physiol Cell Physiol.

[CIT0027] Spruss A, Kanuri G, Uebel K, Bischoff SC, Bergheim I (2011). Role of the inducible nitric oxide synthase in the onset of fructose-induced steatosis in mice. Antioxid Redox Signal.

[CIT0028] Takagawa Y, Berger ME, Hori MT, Tuck ML, Golub MS (2001). Long-term fructose feeding impairs vascular relaxation in rat mesenteric arteries. Am J Hypertens.

[CIT0029] Tappy L, Lê KA (2010). Metabolic effects of fructose and the worldwide increase in obesity. Physiol Rev.

[CIT0030] Török J, Zemančíková A, Tabačeková M (2012). Effect of high-fructose intake on cardiovascular function in normotensive and hypertensive rats. Acta Physiol.

[CIT0031] Török J, Holécyová A, Kyselá S, Bernátová I, Pecháňová O (1998). Changes in reactivity of pulmonary and systemic arteries in chronic NO-deficient hypertension. Cardiol.

[CIT0032] Verma S, Bhanot S, McNeill JH (1994). Antihypertensive effects of metformin in fructose-fed hyperinsulinemic, hypertensive rats. J Pharmacol Exp Ther.

[CIT0033] Yoneyama T, Ohkawa S, Watanabe T, Odamaki M, Kumagai H, Kimura M, Hishida A (1998). The contribution of nitric oxide to renal vascular wall thickening in rats with L-NAME-induced hypertension. Virchows Arch.

[CIT0034] Zemančíková A, Török J (2013). Diminished contractile responses of isolated conduit arteries in two rat models of hypertension. Chin J Physiol.

[CIT0035] Zharikov S, Krotova K, Hu H, Baylis C, Johnson RJ, Block ER, Patel J (2008). Uric acid decreases NO production and increases arginase activity in cultured pulmonary artery endothelial cells. Am J Physiol Cell Physiol.

